# Diphtheria; Some Observations after Inspection of 22,000 School Children for Two Successive Years

**Published:** 1913-01

**Authors:** Claude A. Smith

**Affiliations:** Atlanta, Ga.


### DIPHTHERIA; SOME OBSERVATIONS AFTER INSPECTION OF 22,000 SCHOOL CHILDREN FOR TWO SUCCESSIVE YEARS

By Claude A. Smith, M. D., Atlanta, Ga.

I take this opportunity to submit to the members of the Fulton County Medical Society some observations regarding diphtheria in Atlanta with especial reference to inspection and bacteriolgical examination of inflamed throats. In the Fall of 1910 the Health Department made an inspection of all the children of the public schools and took cultures from the throats of all children which showed signs of inflammation. During the month of October of that year 1500 cultures were taken during this inspection and of these 238 were found to be carrying diphtheroid germs. These 238 cases were kept under quarantine at home, and in December of that year the number of cases reported were less than for the same month of the previous year. As a result of these apparently good returns, in 1911 the Health Department undertook a similar inspection and culture examination of all school children. This was taken up as soon as the schools opened in September. At this time about 23,000 school children were inspected and 1,428 cultures examined and of these 326 were found carrying diphtheriod germs, and of these 155 were from white children and 171 from negro children. The previous year the majority of positive cultures were found among the white children, while in 1911 the positive cultures from the negroes were greater than those from the whites.

On account of the increased number of positive cultures among the negroes, as a test, it was decided to take cultures from every child in one of the negro schools in order to determine what percentage of these children were carrying diphtheroid bacilli. As a result cultures taken from the throats of 440 children showed positive cultures in 121. As most of these children lived in small houses of one and two rooms, it was not possible to quarantine them at home, and the city does not have any contagious hospital for negroes.

The problem was presented to the Board of Health, but for lack of funds and other reasons it was helpless to provide hospital care for these children.

It was suggested that the situation be submitted to the Fulton County Medical Society. This was accordingly done and as the impossibility of quarantining these cases was recognized and as this condition had apparently existed previously, it was decided to modify the then existing strict rules regarding release cultures. By a majority vote, the Fulton County Medical Society recommended to the Board of Health the release of all cases of diphtheria either by one negative culture, or ten days after clinical signs of the disease disappeared. The Board of Health accordingly adopted this suggestion, and for the past twelve months we have been working under this ruling.

During the year 1912 (January 1st to December 1st) 234 positive cultures have been sent to the laboratory by physicians and of these 21 have died. The mortality in 1910 was 15, in 1911, 17. You will thus see that there has been a slight increase in the number of deaths. In this short time we were able to gain some idea of just how much we have been accomplishing by our so-called home quarantine. At best this quarantine is rarely thorough and among the poorer classes is an impossibility, and especially during the past 12 monhts, on account of the ruling suggested by the Fulton County Medical Society and adopted by the Board of Health, the quarantine has been less efficient and yet the mortality ha sincreased but slightly. Also, it is barely possible that this mortality increase might be partially accounted for by the increased population and increased congestion.

It is interesting to note that of the 238 children found in the schools durng 1910 with positive cultures from their throats,

14 of these same children were again found wit hthe diphtheroid germs in their throats in 1911. It would be interesting to know whether these 14 children were harboring the germs from year to year.

The question as to the virulence of the germs is something to be considered. Practically all of the different types of Westbrook were recognized, but our clinical observations have not afforded much information as to virulence. In some instances it has been observed that the apparently virulent organism producing a fatal result in one child was of almost no consequence in the throats of other children. According to Sahli and others this test is of far more value than laboratory tests on animals.

In our work we have much to indicate that diphtheria is a mild disease and the malignant cases few and far between. Children associated with malignant cases rarely themselves develop severe forms of the disease. Resistance of the individual appears to us to play a greater part than the virulence of the organism. Much time and study have been given to virulence of organism but comparatively little to natural resistance. It appears to me that the greatest good to be accomplished and the greatest possibility of accomplishment lies in the direction of increasing resistance. Our meager observations allong this line indicate that resistance can be increased with very little effort and at a small cost.

I would call your attention to the fact that the number of cases of diphtheria reported by physicians has not varied much during the past three years. In 1910 they reported 252 cases, in 1911, 265 cases, and in 1912 for eleven months 234 cases.

The school insection disclosed the fact that many of the children carrying diphtheroid germs gave histories showing that they had passed through moderately severe attacks of diphtheria without the attendance of a physician. It appeared that more cases of diphtheria were treated at home without being recognized than reported as diphtheria to the Health Department.

In the school inspection where cultures were taken from all inflamed throats only a small per cent. were found carrying diphtheroid germs. I mention this to refute the idea which is prevalent that it is possible to find diphtheroid germs in almost any healthy or inflamed throat. Our investigations indicate that diphtheroid germs are rapidly thrown off by healthy throats. Also, it was possible in many instances to trace the throat infection to close association with other children already infected.

*Summary.*—Apparently only a small number of the cases of true diphtheria are recognized and reported to the Health Department to be placarded.

Where these cases are not recognized and quarantined there is no violent spread of the disease.

Even where cases are recognized and placarded, proper quarantine is often impossible.

Raising the resistance of children is more to be desired than quarantine.

On a whole, diphtheria, at present in Atlanta, appears to be a mild disease and the malignant form is an exception.

Diphtheroid germs appear to spread most commonly as the result of close association of individuals.

